# RadiSurg—Need of Implementation of an Interdisciplinary Surgical-Based Radiological Anatomy Course

**DOI:** 10.1007/s40670-025-02492-8

**Published:** 2025-09-09

**Authors:** Nora Corinna Altmayer, Elias Khajeh, Verena Steinle, Anna Lintner, Johanna Fellhofer-Hofer, Felix Nickel, Arianeb Mehrabi, Fee Klupp

**Affiliations:** 1 Department of General, Visceral and Transplantation Surgery, University Hospital Heidelberg, Im Neuenheimer Feld 420, Heidelberg, 69120 Germany; 2 Department of General, Visceral, Vascular and Pediatric Surgery, Saarland University Hospital, Kirrberger Str. 100, Homburg, 66421 Germany; 3 Department of Diagnostic and Interventional Radiology, University Hospital Heidelberg, Im Neuenheimer Feld 420, Heidelberg, 69120 Germany; 4 Department of General, Visceral and Thoracic Surgery, University Medical Center Hamburg–Eppendorf, Martinistr. 52, Hamburg, 20246 Germany

**Keywords:** Interdisciplinary education, Final year students, Anatomy-surgery-radiology course, Multimodal teaching, Expanded competencies

## Abstract

**Introduction:**

At the beginning of medical school, anatomy courses usually take place on cadavers. Mostly several years of medical training without any further clinically applied anatomical lessons are following. Therefore, we decided to review and refresh students’ knowledge of anatomy with special regard to surgical clinical settings.

**Methods:**

An interdisciplinary surgical-based radiology seminar for final year students in their internship year and fourth year students during their surgical semester was performed starting with a surgical-radiological-anatomical knowledge exam. Afterwards, clinical cases were discussed in detail including for example videos from operations and corresponding radiological imaging by specialists from both surgery and radiology. At the end of the seminar, the same knowledge survey and an evaluation questionnaire were carried out.

**Results:**

Both final year students and fourth year students showed a significant increase regarding the knowledge questions after the interactive interdisciplinary seminar ( *p* 0.001). In addition, fourth year student’s post-seminar results were significantly higher than post-seminar results reached by final year students ( *p* 0.001). Ninety-seven percent of final year students perceived the seminar as useful preparation for the first year as a resident.

**Conclusion:**

In summary, this study demonstrates the value of implementing a clinically applied anatomy refresher into the medical curriculum to increase students’ knowledge and better prepare them for their residency.

## Introduction

 At the beginning of medical school, anatomy courses usually take place without clinical correlation [1]. Commonly, this is followed by several years of medical training without teaching clinically applied anatomy. As cadaver donors are difficult to obtain, other clinically applied anatomy teaching approaches are required and necessarily needed [2]. Therefore, online computer courses or 3D models are increasingly integrated into medical studies in order to deepen knowledge about the human body and make it easier to understand [3, 4]. Moreover, virtual dissection tables are sometimes used in modern anatomy curricula to support cadaver-based methods as well as to improve anatomical-based clinical knowledge [5]. Nevertheless, interdisciplinary lessons focusing on surgically applied anatomy are not usually included in current medical curricula and studies on the subject of surgical anatomical courses with radiological references are scarce. However, students evaluated the implementation of an interdisciplinary curriculum consisting of radiation oncology, radiology, and nuclear medicine positively [6]. Furthermore, Zhao et al. demonstrated the effectiveness of an online teaching lesson consisting of radiological case reports with pathological correlations [7].

 Final year medical students often feel relegated being “only hook-holders” during their time in the department of surgery in their internship year [8]. It is noteworthy that during the course of medical studies many students decide against specialist training in surgery. One reason for the unattractiveness of the surgical specialty among young surgeons could be the working condition, rather than surgery itself [9]. This might be due to a lack of planning certainty, uneven work-life balance during everyday clinical practice, and limited establishment opportunities. A further reason for deciding against surgical residency may be the absence of practical training during the course of medical studies [10]. In the USA, a post-Flexnerian reform of initially theoretical-based medical education is required [11]. Subsequently, new interdisciplinary and cognitive skills are required in order to better adapt medical studies to the challenges of medical profession [12].

Due to a long break between pure anatomy and clinical medical courses, the rationale for our study was to compare existing radiological anatomical knowledge of fourth and final year medical students using clinical cases on a surgical basis. In addition, an assessment questionnaire was used to assess possible backgrounds of how medical students fare. One aim of the study was to encourage interdisciplinary thinking. Another aim was to find out how medical studies can be optimized to better prepare students for the future challenges of the medical profession.

## Materials and Methods

### Participants and Setting

The participants were medical students enrolled at the University of Heidelberg. The medical degree program lasts a total of 6 years, the first five of which consist of theoretical and practical courses. State examinations are held after 2 years and after 5 years. After the second state examination, students complete their practical year, which consists of three tertials of surgery, internal medicine, and an elective subject.

 There were two groups of participants: First, medical students in their final year of study, the so-called practical year or internship year, were included. In Germany, medical studies are lasting 6 years of which the first 5 years are mainly theoretical in content. The last year after the written final exam is practical. These final year students passed their written state examination. Afterwards they have to spend one of three tertials in the surgery department at the time of the study. The three tertials consist of a mandatory tertial in internal medicine and surgery, as well as an elective subject. Each tertial lasts 16 weeks. These students will have to pass an oral exam at the end of the third tertial (*n*_before_ = 38, *n*_after_ = 41). The second group of participants were medical students in their fourth year of study (*n*_before_ = 38, *n*_after_ = 29) during the surgical training block. Participation in this study was voluntary and anonymous.

During the surgical tertial in the final year of training, medical students must gain experience in general surgery and visceral surgery for at least 8 weeks up to 16 weeks. Other possible surgical departments like vascular or trauma surgery can be chosen too for at least 4 weeks up to 8 weeks. During this time, students take part in ward rounds, support the surgeons during an operation, learn how to write medical letters, and present patients in the surgery planning meeting.

Whereas, in the fourth year of medical training at the University of Heidelberg, students complete weekly courses in vascular surgery, cardiac surgery, thoracic surgery, orthopedics and trauma surgery, visceral surgery, urology, and emergency medicine. During this time, students learn practical skills such as suturing porcine skin or surgical knotting. Anamnesis interviews with subsequent diagnosis are learnt with the help of acting patients. Emergency medical treatment procedures are also learnt on simulation dolls. In the subject “clinical-pathological conference,” fourth year medical students learn to link surgical clinical pictures with histological images and radiological images.

### Study Design, Procedure, and Statistical Analysis

 The “Radisurg ” seminar began with a knowledge test (*n* = 32 points maximum) including surgical knowledge questions from different subject areas with reference to radiological imaging (e.g., hollow organ perforation, hepatocellular carcinoma, perforated cholecystitis) with resulting therapies. The questions included both KPrim-choice questions and single-choice questions. The response time per question was 90 s. Afterwards specialists from the departments of surgery and radiology discussed requested basic surgical topics in detail, including operation photos, operation videos, and the corresponding imaging (CT, MRI, angiography, X-ray, ultrasound). The session lasted 60 min, followed by the same surgical knowledge questionnaire that was answered in the beginning. After the knowledge test, nine evaluation questions followed. The evaluation questions were analyzed using a 5-point Likert scale [13]. A Likert scale consists of five statements that all measure the same characteristic. This allows the assessment of agreement or disagreement; for example, a Likert score of 1 means that the participant strongly agrees or strongly disagrees. If a Likert score of 1 means that the participant strongly agrees, then a Likert score of 2 means that the participant agrees less than with Likert scale 1, but more than with Likert scale 3. Students who did not respond to the questionnaires were not included in the study.

 Statistical tests were carried out using Excel™ 2019, SPSS™ (version 29), and GraphPadPrism™ (version 9). Results of the knowledge questions were reported as box plots with 25th–75th percent quartiles with interquartile range and minimum/maximum. To calculate differences between independent samples, the Mann–Whitney *U* test was assessed. The chi-square test and Fisher’s exact test were performed for evaluation questions. A *p* -value ≤ 0.05 was considered significant. Percentages of numerical Likert scale of 1 and 2 were added together to receive a statement about the positive evaluations. Congruently, to get a statement about the negative ratings, the percentages of the scale of 4 and 5 were summed up accordingly. Because some Likert scale ranges were not chosen by the students, some evaluation questions could not be calculated.

### Ethics Approval

Ethics approval was granted by the University of Heidelberg (S-334/2023). This study was performed in line with the principles of the Declaration of Helsinki. Written informed consent was obtained from all individual participants included in the study.

## Results

### Knowledge Questions

The study included 76 students before the seminar. All of them completed the knowledge test. After the seminar, 69 students passed the same test. Differences in the number of participants before and afterwards were due to voluntary participation in the knowledge test. Therefore, there was no duty to submit the knowledge test or the evaluation questionnaire.

**Fig. 1:**
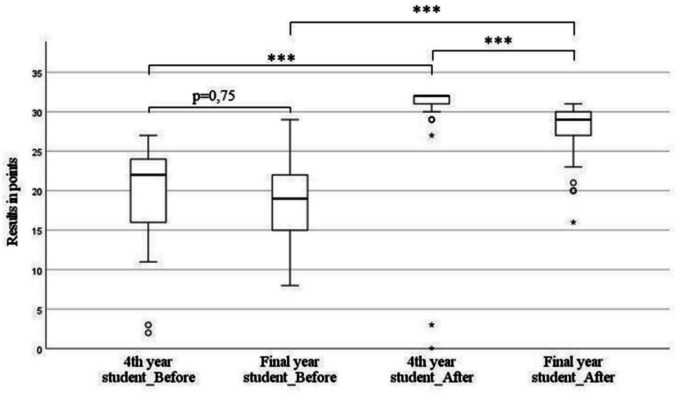
Test results in points before and after the course. The total result was 32 points. Bar represents median + interquartile range (fourth year: *n*_before_ = 38, *n*_after_ = 29, final year: *n*_before_ = 38, *n*_after_ = 41)

 Final year students reached a median of 19 points (*n* = 38) before and 29 points (*n* = 41) after the seminar. Fourth year students achieved a median of 22 points as well as fourth year medical students (*n* = 38) before and 32 points (*n* = 29) afterwards. Both groups, final and fourth year students, received a significant increase in knowledge after the course (*p* = 0.001, Fig. 1).

Fourth year students achieved a better result before the course compared to final year students; however, this failed to be significant, but after the seminar, fourth year students reached significant higher results compared to last year students during their practical year. The mean scores of final year students improved by 29.85%, while those of fourth year students increased by 29.53%.

### Results of the Evaluation Questionnaire

**Fig. 2:**
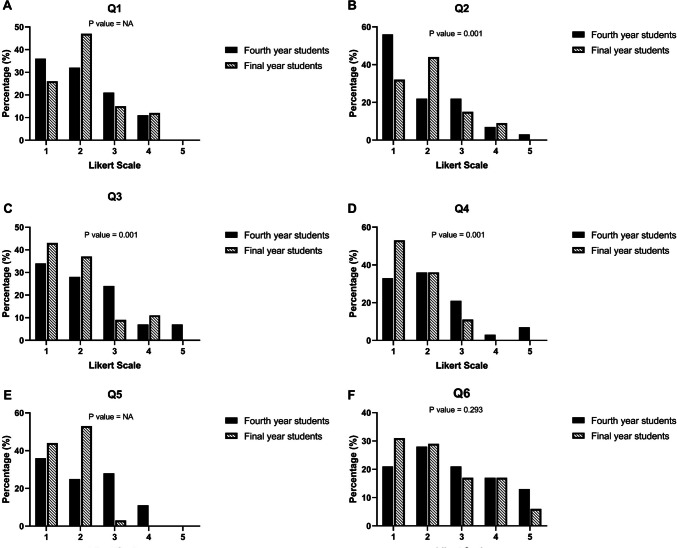

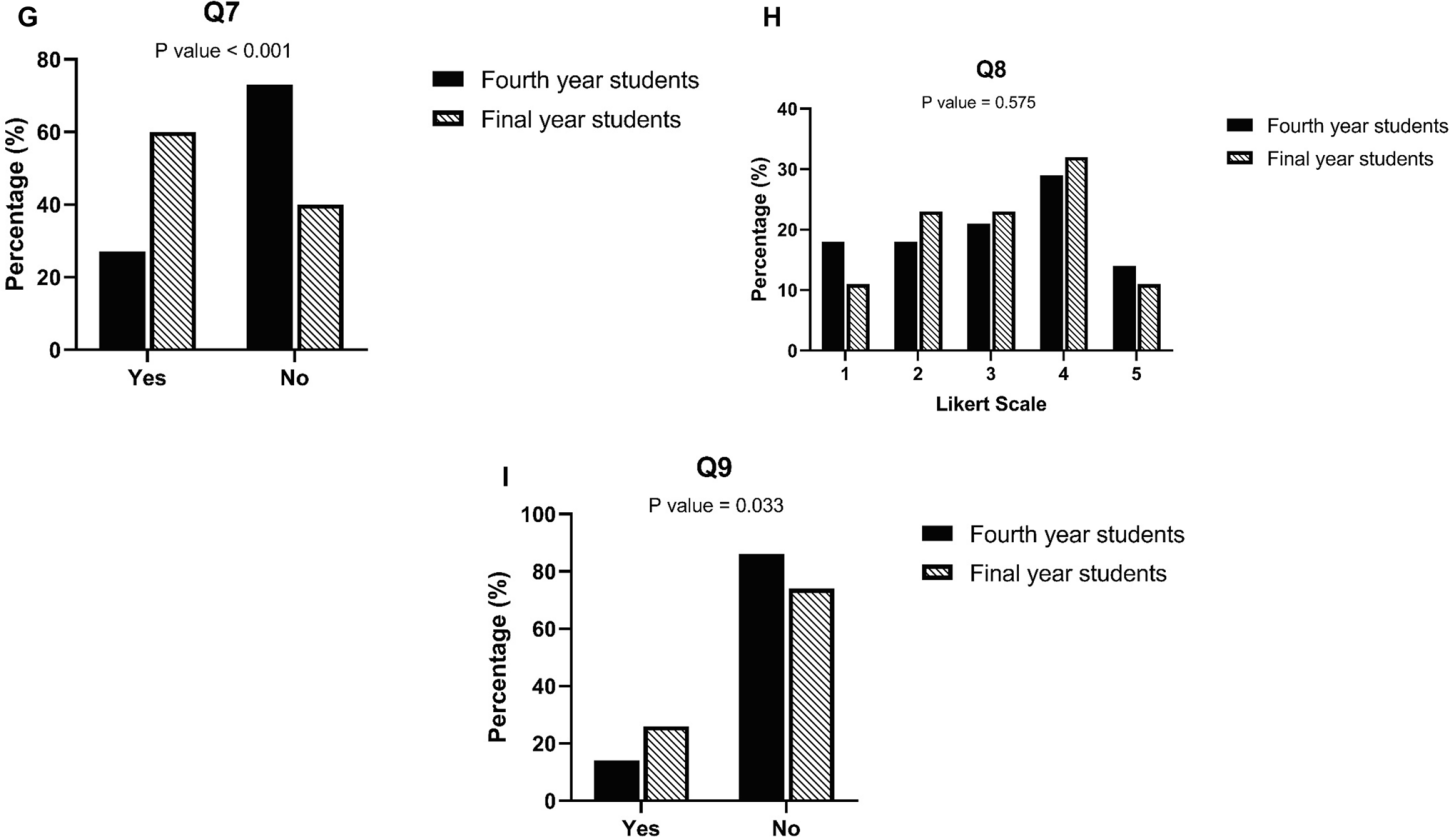
**a** Evaluation question Q1: “Was anatomical knowledge repeated during surgery courses in medical studies?” (*n*_fourth year_ = 28, *n*_final year_ = 34, Likert scale 1: *n*_fourth year_ = 10, *n*_final year_ = 9, Likert scale 2: *n*_fourth year_ = 9, *n*_final year_ = 16, Likert scale 3: *n*_fourth year_ = 6, *n*_final year_ = 5, Likert scale 4: *n*_fourth year_ = 3, *n*_final year_ = 4, Likert scale 5: *n*_fourth year_ = 0, *n*_final year_ = 0). **b** Evaluation question Q2: “Was anatomical knowledge repeated during radiology courses in medical studies?” (*n*_fourth year_ = 28, *n*_final year_ = 34, Likert scale 1: *n*_fourth year_ = 13, *n*_final year_ = 11, Likert scale 2: *n*_fourth year_ = 6, *n*_final year_ = 15, Likert scale 3: *n*_fourth year_ = 6, *n*_final year_ = 5, Likert scale 4: *n*_fourth year_ = 2, *n*_final year_ = 3, Likert scale 5: *n*_fourth year_ = 1, *n*_final year_ = 0). **c** Evaluation question Q3: “Do you feel like your anatomical knowledge is linked with surgical knowledge and radiology knowledge?” (*n*_fourth year_ = 29, *n*_final year_ = 35, Likert scale 1: *n*_fourth year_ = 10, *n*_final year_ = 15, Likert scale 2: *n*_fourth year_ = 8, *n*_final year_ = 13, Likert scale 3: *n*_fourth year_ = 7, *n*_final year_ = 3, Likert scale 4: *n*_fourth year_ = 2, *n*_final year_ = 4, Likert scale 5: *n*_fourth year_ = 2, *n*_final year_ = 0). **d** Evaluation question Q4: “I think the seminar will be useful as preparation for the oral exam.” (*n*_fourth year_ = 28, *n*_final year_ = 28, Likert scale 1: *n*_fourth year_ = 10, *n*_final year_ = 15, Likert scale 2: *n*_fourth year_ = 8, *n*_final year_ = 10, Likert scale 3: *n* _fourth year_ = 7, *n*_final year_ = 3, Likert scale 4: *n*_fourth year_ = 2, *n*_final year_ = 0, Likert scale 5: *n*_fourth year_ = 2, *n*_final year_ = 0). **e** Evaluation question Q5: “I think the seminar will be useful as preparation for the first year of residency.” (*n*_fourth year_ = 28, *n*_final year_ = 28, Likert scale 1: *n*_fourth year_ = 10, *n*_final year_ = 14, Likert scale 2: *n*_fourth year_ = 7, *n*_final year_ = 17, Likert scale 3: *n*_fourth year_ = 8, *n*_final year_ = 1, Likert scale 4: *n*_fourth year_ = 3, *n*_final year_ = 0, Likert scale 5: *n*_fourth year_ = 0, *n*_final year_ = 0). **f** Evaluation question Q6: “I am interested in surgery.” (*n*_fourth year_ = 29, *n*_final year_ = 35, Likert scale 1: *n*_fourth year_ = 6, *n*_final year_ = 11, Likert scale 2: *n*_fourth year_ = 8, *n*_final year_ = 10, Likert scale 3: *n*_fourth year_ = 6, *n*_final year_ = 6, Likert scale 4: *n*_fourth year_ = 5, *n*_final year_ = 6, Likert scale 5: *n*_fourth year_ = 4, *n*_final year_ = 2). **g** Evaluation question Q7: “Do you have already undertaken clinical internships in surgery?” (*n*_fourth year_ = 26, *n*_final year_ = 35, yes: *n*_fourth year_ = 7, *n*_final year_ = 21, no: *n*_fourth year_ = 19, *n*_final year_ = 14). **h** Evaluation question Q8: “I am interested in radiology.” (*n*_fourth year_ = 28, *n*_final year_ = 35, Likert scale 1: *n*_fourth year_ = 5, *n*_final year_ = 4, Likert scale 2: *n*_fourth year_ = 5, *n*_final year_ = 8, Likert scale 3: *n*_fourth year_ = 6, *n*_final year_ = 8, Likert scale 4: *n*_fourth year_ = 8, *n*_final year_ = 11, Likert scale 5: *n* _fourth year_ = 4, *n*_final year_ = 4). **i** Evaluation question Q9: “Do you have already undertaken clinical internships in radiology?” (*n*_fourth year_ = 28, *n*_final year_ = 35, yes: *n*_fourth year_ = 4, *n*_final year_ = 9, no: *n*_fourth year_ = 24, *n*_final year_ = 26)

 We collected evaluation questionnaire responses after the seminar (Table 1). Seventy-three percent of final year students stated that anatomical knowledge was repeated in surgical seminars during their medical studies (Question 1 = Q1, Fig. 2 a, p = NA [NA = not analyzable]), respectively 76% during radiological seminars (Question 2 = Q2, Fig. 2 b, p = 0.001). Sixty-two percent of fourth year students and 80% of final year students understand that the medical disciplines anatomy, surgery, and radiology are closely linked to each other (Question 3 = Q3, Fig. 2 c, p = NA). Sixty-nine percent of fourth year students stated that the seminar was helpful in preparing for the oral exam, 89% of final year students saying the same (Question 4 = Q4, Fig. 2 d, p = 0.001). The seminar was evaluated by 61% of the fourth year and by 97% of final year students as a useful preparation for the first year as a resident (Question 5 = Q5, Fig. 2 e, p = 0.001). The question whether one is interested in surgery was answered positively by only 49% of fourth year students, respectively, by 60% of final year students (Question 6 = Q6, Fig. 2 f, p = 0.293). Twenty-seven percent of fourth year students and 60% of final year students have already undertaken clinical internship in surgery (Question 7 = Q7, Fig. 2 g, p = 0.001). Thirty-six percent of fourth year students and 34% of final year students were interested in radiology (Question 8 = Q8, Fig. 2 h, p = 0.575). Fourteen percent of fourth year students and 26% of final year students have already completed clinical internship in radiology (Question 9 = Q9, Fig. 2 i, p = 0.033).

**Table 1:** Results of the evaluation questionnaire

				Likert scale				
				1	2	3	4	5
Evaluation question Q1	“Was anatomical knowledge repeated during surgery courses in medical studies?”	Fourth year students	*n* = 28	36% (*n* = 10)	32% (*n* = 9)	21% (*n* = 6)	11% (*n* = 3)	0% (*n* = 0)
		Final year students	*n* = 34	26% (*n* = 9)	47% (*n* = 16)	15% (*n* = 5)	12% (*n* = 4)	0% (*n* = 0)
Evaluation question Q2	“Was anatomical knowledge repeated during radiology courses in medical studies?”	Fourth year students	*n* = 28	46% (*n* = 13)	22% (*n* = 6)	22%(*n* = 6)	7% (*n* = 2)	3% (*n* = 1)
		Final year students	*n* = 34	32% (*n* = 11)	44% (*n* = 15)	15% (*n* = 5)	9% (*n* = 3)	0% (*n* = 0)
Evaluation question Q3	“Do you feel like your anatomical knowledge is linked with surgical knowledge and radiology knowledge?”	Fourth year students	*n* = 29	34% (*n* = 10)	28% (*n* = 8)	24% (*n* = 7)	7% (*n* = 2)	7% (*n* = 2)
		Final year students	*n* = 35	43% (*n* = 15)	37% (*n* = 13)	9% (*n* = 3)	11% (*n* = 4)	0% (*n* = 0)
Evaluation question Q4	“I think the seminar will be useful as preparation for the oral exam.”	Fourth year students	*n* = 28	33% (*n* = 8)	36% (*n* = 10)	21% (*n* = 6)	3% (*n* = 1)	7% (*n* = 2)
		Final year students	*n* = 28	53% (*n* = 15)	36% (*n* = 10)	11% (*n* = 3)	0% (*n* = 0)	0% (*n* = 0)
Evaluation question Q5	“I think the seminar will be useful as preparation for the first year of residency.”	Fourth year students	*n* = 28	36% (*n* = 10)	25% (*n* = 7)	28% (*n* = 8)	11% (*n* = 3)	0% (*n* = 0)
		Final year students	*n* = 28	44% (*n* = 14)	53% (*n* = 17)	3% (*n* = 1)	0% (*n* = 0)	0% (*n* = 0)
Evaluation question Q6	“I am interested in surgery.”	Fourth year students	*n* = 29	21% (*n* = 6)	28% (*n* = 8)	21% (*n* = 6)	17% (*n* = 5)	13% (*n* = 4)
		Final year students	*n* = 35	31% (*n* = 11)	29% (*n* = 10)	17% (*n* = 6)	17% (*n* = 6)	6% (*n* = 2)
				Yes	No			
Evaluation question Q7	“Do you have already undertaken clinical internships in surgery?”	Fourth year students	*n* = 26	27% (*n* = 7)	73% (*n* = 19)			
		Final year students	*n* = 35	60% (*n* = 21)	40% (*n* = 14)			
				1	2	3	4	5
Evaluation question Q8	“I am interested in radiology.”	Fourth year students	*n* = 28	18% (*n* = 5)	18% (*n* = 5)	21% (*n* = 6)	29% (*n* = 8)	14% (*n* = 4)
		Final year students	*n* = 35	11% (*n* = 4)	23% (*n* = 8)	23% (*n* = 8)	32% (*n* = 11)	11% (*n* = 4)
				Yes	No			
Evaluation question Q9	“Do you have already undertaken clinical internships in radiology?”	Fourth year students	*n* = 28	14% (*n* = 4)	86% (*n* = 24)			
		Final year students	*n* = 35	26% (*n* = 9)	74% (*n* = 26)			

### Short Summary of Results

Final and fourth year students received a significant increase in knowledge after the course. Sixty-two percent of fourth year students and 80% of final year students understand that the medical disciplines anatomy, surgery, and radiology are closely linked to each other. The seminar was evaluated by 61% of the fourth year and by 97% of final year students as useful preparation for the first year as a resident.

## Discussion

 The purpose of the current study was to investigate the level of knowledge regarding clinical anatomy in the fields of surgery and radiology in final medical students during their practical year after their state examination versus fourth year medical students during their surgical semester. This educational model aimed to better combine advanced, surgically and radiologically relevant anatomy. We could demonstrate that both final year and fourth year students reached significant better results in the knowledge tests after the lesson. Fourth year student’s post-seminar results were significantly higher than the post-seminar results achieved by final year students. Even in the pre-seminar test, fourth year students reached better results than the final year students. Actually, one would assume that final year students during their practical year shortly before their final exam and medical approbation should have achieved better results than their fourth year colleagues. It is tempting to speculate, if this could be attributable to the COVID pandemic, where less or almost no practical lessons were held resulting in a lack of clinical experience [14]. Final year students were particularly more affected by this, meanwhile fourth year students received more hands-on learning and are exposed less to COVID burden because restrictions were shrinking. A scoping review dealing with medical academic performance including 24 publications explored three studies showing that students achieved lower grades during COVID-19 pandemic [15–18]. Another reason may be that the fourth year students are closer in time to the anatomy course, which regularly takes place at the beginning of the medical degree program. Final year students may have partially forgotten the surgical content due to a lack of repetition, and their anatomy course was at least 6 years ago. Farley et al. also propagated that the level of anatomy instruction must be maintained in the early years of medical education and that instruction in advanced anatomy such as endoscopic, endovascular, and advanced imaging techniques should be intensified in later years [19].

 Regarding the evaluation questions, 97% of final year students stated the course as useful preparation for the first year as a resident. Performing surgical approaches with the secondary benefit of practicing basic surgical techniques could be a future broadening, especially for those students who want to start a career as surgical residents. Ferlauto et al. and Dee et al. treat of educational models that deal with advanced, surgically relevant anatomy in medical school, partly by performing realistic surgical procedures on anatomical donors [20, 21]. Dee et al. performed a procedure-oriented surgical anatomy class for third and fourth year medical students by teaching procedures as left nephrectomy, first rib resection, and carotid endarterectomy. Carmichael et al. created a cadaver-based seminar cofacilitated by anatomists and surgical residents for medical students in an early stage of medical education to provide clinical context for their learning and to introduce positive surgical role models early during medical training [22]. Immersive virtual reality (IVR) might be a safe and effective training tool to achieve a course expansion, too [23]. Parkhomenko et al. released a study in which IVR led to an alteration of percutaneous nephrolithotomy approaches in 40% of cases [24]. IVR has been used for monthly lectures in interventional radiology for medical students as well [25]. But a comparison with a control group was not carried out. Another improvement could be the integration of a 360° virtual reality. In pilot studies, medical students and surgical residents self-reported that watching the 360° videos led to an improvement of self-efficacy. Students stated that they felt the videos improved their learning outcomes [26]. IVR has improved the performance of procedures by surgical trainees and surgeons in visceral and vascular surgery [27, 28]. It is plausible that this effort could also be transferred in medical education.

 Dettmer et al. demonstrated that second year students gained advantages from a course combining anatomy, surgery, and radiology using cadavers. Their motivation levels were higher than average, and the students affirmed that their personal understanding of anatomy had improved. In addition, interest in plastic surgery and anatomy of the hand as well as radiologic anatomy was increased after the seminar and students performed very well in the test conducted at the end of the course. Although the course was offered early in medical studies, there was no continuity in terms of follow-up courses [29]. Bradley et al. performed a study with 29 assistant doctors in their first year of residency. After completing a course that included anatomy, surgery, and radiology with surgical units, cadaver dissections, and seminars, all participants felt that their knowledge had increased, and 58.62% of them reported an enhancement in their clinical skills. Average knowledge rating before and after the course increased from 55 to 81%, which corresponds to an improvement of approximately 30%, which is in line with our results [30].

 Our study demonstrates that final year students achieved 56.25% in the knowledge query in the pre-seminar test. Contrary to this result, over 70% of the final year students think that they received enough repetition regarding clinically applied surgical radiology during their medical education. Until now, longitudinal learning of interdisciplinary skills is only a small part of medical training. So contrary to the expectation, many students—62% of fourth year students and 80% of final year students—stated that their knowledge seemed to be interdisciplinary connected. However, the results before the seminar in last year’s students were not sufficient; fourth year students reached a result of 68.75%. Mostly medical students have to achieve a minimum percentage of 60% right answers in order to pass an exam. Nevertheless, final year students only achieved an average of 56.81% of the total points which would have resulted in 22 of 32 students failing the exam. The addition of asynchronous learning formats like online courses might be an option to further increase performance, offering e.g. a preceding online basic lecture [31]. Beyond that, an additional educational approach with live stream videos before the seminar might lead to a gain of knowledge [32].

 As limitations of our study should be mentioned that, due to completely anonymized data of the study participants, no pairwise statistical analysis could be calculated for the knowledge query. Additionally, the evaluation questionnaire was not carried out before and after the seminar. This is the reason why we cannot show that participation in the seminar increases, decreases, or changes interest in surgery or radiology. Further studies with more study participants have to investigate the sustainability of such a surgical-based anatomy refresher. Long-term follow-up questionnaires should be assessed to evaluate the learning progress over time and to assess how well the conveyed content is retained by the participants.

## Conclusions

This study gives a hint regarding the meaningfulness of a surgery-radiology seminar in the curriculum of medical education in order to refresh clinical applied anatomical knowledge. Consequently, students may be better prepared for professional life in different medical disciplines. Further studies with more participants and measurements of long-term outcomes are needed to better analyze and evaluate interdisciplinary courses. We deduce that clinically applied surgical radiology lessons should be integrated into the medical curriculum in a longitudinal way to achieve a sustainable learning effect.
